# The quality, safety and governance of telephone triage and advice services – an overview of evidence from systematic reviews

**DOI:** 10.1186/s12913-017-2564-x

**Published:** 2017-08-30

**Authors:** Rebecca Lake, Andrew Georgiou, Julie Li, Ling Li, Mary Byrne, Maureen Robinson, Johanna I. Westbrook

**Affiliations:** 1 Australian Digital Health Agency, Level 25, 56 Pitt Street, Sydney, NSW 2000 Australia; 2 Centre for Health Systems and Safety Research, Australian Institute of Health Innovation, Macquarie University, Level 6, 75 Talavera Road, Sydney, NSW 2109 Australia; 3 Healthdirect Australia, Level 19, 133 Castlereagh Street, Sydney, NSW 2000 Australia

**Keywords:** After-hours care, General practice, Primary health care, Telephone triage, Tele-consultation

## Abstract

**Background:**

Telephone triage and advice services (TTAS) are increasingly being implemented around the world. These services allow people to speak to a nurse or general practitioner over the telephone and receive assessment and healthcare advice. There is an existing body of research on the topic of TTAS, however the diffuseness of the evidence base makes it difficult to identify key lessons that are consistent across the literature. Systematic reviews represent the highest level of evidence synthesis. We aimed to undertake an overview of such reviews to determine the scope, consistency and generalisability of findings in relation to the governance, safety and quality of TTAS.

**Methods:**

We searched PubMed, MEDLINE, EMBASE, CINAHL, Web of Science and the Cochrane Library for English language systematic reviews focused on key governance, quality and safety findings related to telephone based triage and advice services, published since 1990. The search was undertaken by three researchers who reached consensus on all included systematic reviews. An appraisal of the methodological quality of the systematic reviews was independently undertaken by two researchers using A Measurement Tool to Assess Systematic Reviews.

**Results:**

Ten systematic reviews from a potential 291 results were selected for inclusion. TTAS was examined either alone, or as part of a primary care service model or intervention designed to improve primary care. Evidence of TTAS performance was reported across nine key indicators – access, appropriateness, compliance, patient satisfaction, cost, safety, health service utilisation, physician workload and clinical outcomes. Patient satisfaction with TTAS was generally high and there is some consistency of evidence of the ability of TTAS to reduce clinical workload. Measures of the safety of TTAS tended to show that there is no major difference between TTAS and traditional care.

**Conclusions:**

Taken as a whole, current evidence does not provide definitive answers to questions about the quality of care provided, access and equity of the service, its costs and outcomes. The available evidence also suggests that there are many interactional factors (e.g., relationship with other health service providers) which can impact on measures of performance, and also affect the external validity of the research findings.

**Electronic supplementary material:**

The online version of this article (10.1186/s12913-017-2564-x) contains supplementary material, which is available to authorized users.

## Background

The provision of after-hours primary medical care services has been changing in many countries in response to increasing demand and pressures on existing services [1–3]. Telephone triage and advice services (TTAS) are one approach to expanding the provision of out-of-hours medical care. TTAS involves people with a health problem receiving assessment and advice over the telephone [4]. TTAS advice may include a recommendation to visit an Emergency Department (ED), make an appointment with a general practitioner (GP) or other healthcare provider, or the administration of home or self-care [5, 6]. The most common TTAS delivery models are either nurse- or physician-led, very often involving the use of call centre technology, incorporating clinical decision support systems to aid in the evaluation of patients’ health conditions [7].

One of the major drivers for TTAS has been the need to reduce the burden on GPs and EDs. Some estimates suggest that more than half of out-of-hours calls can be handled by telephone advice alone [8]. With this in mind it would seem reasonable to assume that access to health information and advice could relieve pressures on GP services and EDs particularly through reductions in “inappropriate” or “avoidable” attendances [9–11]. For many countries TTAS has become an important part of the organisation and delivery of out-of-hours care [12]. A number of healthcare call centres have been established in the United Kingdom (UK) [13], Australia, Sweden, Denmark, the Netherlands, Canada, along with the United States of America (USA), which does not have government-sponsored national telephone triage access but does have some localised systems [6, 11, 14].

With the expansion of TTAS over the last decade there has been a corresponding increase in its popularity and utilisation across many jurisdictions [11, 15]. This growth has in turn stimulated interest in the potential to incorporate WebRTC (real time communications) technologies [16], to provide accessible and easy-to-use video capabilities to expand the type of advice and care provided. These capabilities may have the potential to address the rising demands of over stretched health services [13], and enable access for people living in remote areas. TTAS involve many medical, technological and social/organisational challenges [17, 18] around how the service is run and monitored [19], along with issues related to the service’s accessibility, quality and appropriateness [20, 21]. The challenges and opportunities afforded by major telehealth care innovations underscore the need for a comprehensive assessment of available evidence related to their quality, safety and governance.

The existing evidence base on the topic examined aspects of TTAS from different perspectives (e.g., clinical, management and patients) using a wide range of measures that are not consistently applied or assessed. This makes it difficult for policymakers to use existing evidence to inform policy decisions. Systematic reviews represent the highest level of evidence synthesis. We aimed to undertake an overview of such reviews to determine the scope, consistency and generalisability of findings in relation to the governance, safety and quality of TTAS [22].

## Methods

### Inclusion criteria

Our overview included any systematic review focused on telephone based triage and advice services, available in English; and published since 1990. We decided to commence our search at 1990 as the types of telehealth services under consideration commenced operation around this time [1].

We excluded reviews which were related only to the use of telephone advice for a specific population or demographic (e.g. ethnic minority), condition (e.g., depression) medical specialty (e.g., asthma); and ongoing or chronic conditions (e.g., diabetes); technical assessments not related to patient or healthcare outcomes; and general health education. Table 1 lists the inclusion and exclusion criteria used in this overview.

**Table 1:** Inclusion and exclusion criteria

Inclusion Criteria	Exclusion Criteria
Systematic reviews (eg. rapid reviews/evidence scans, meta-analyses)	Original studies, trials, non-systematic reviews (e.g., literature reviews)
Full text available	Abstract only; full text not available
Publication date: 1990 - current	Pre-1990
Available in English	Not available in English
Related to general, primary care	E-mail or video communication
Examined telephone-based triage and GP consultation or out-of-hours primary care models that included TTAS	Clinician to clinician communication Technical assessments
No specific population or demographic	Population/demographic specific Condition or disease specific, or specific to a particular medical specialty (e.g., diabetes)
	Related only to health education, patient monitoring or case management

To maximise our search criteria we used the PICO (Population, Intervention, Comparison, Outcome) search strategy form. The patient or problem targeted were presenting patients, requiring triage or consultation. The intervention was telephone based triage, advice or consultation by telephone. Most of the studies included in the reviews compared telephone advice with face-to-face consultations, but some also compared telephone advice given by different healthcare professionals. Key indicators included those performance measures related to the safety, quality or governance of TTAS and/or other relevant patient/health-related outcomes.

### Search methods

We sourced our results from the following databases: PubMed, MEDLINE, EMBASE, CINAHL, Web of Science and the Cochrane Library. The search terms used included ‘tele’ terms, with ‘tele’ truncated to elicit all related terms (telehealth, telemedicine, telephone, etc.); terms relating to the type of care: triage, advice, consultation; and other related terms such as after-hours, out-of-hours and primary care. The full search strategy is outlined in Additional file 1.

The title and abstract of the reviews identified through our searches was scrutinised, and potentially relevant papers were downloaded for further consideration. Following the search, the full text of these reviews was examined and those which fit our criteria were added to our results. We also scrutinised the reference lists of all relevant papers to identify reviews for inclusion or exclusion.

To ensure reliability, the search strategy was devised by three researchers (AG, LL and RL), the search was completed by one team member (RL) and then replicated by another (LL), and all three researchers discussed the identified texts to arrive at a consensus on the reviews for inclusion.

### Data collection and analysis (selection, data extraction and management)

The final group of texts was read in full and the data extracted. We abstracted descriptive details of the included systematic reviews: the authors, title, year published and country. Data were collected about the studies reviewed in each text: the number and type of reviewed studies, the date range of the studies and the study populations (Table 1). Information was compiled about the indicators or performance measures used, the purpose of the review, the design, search methods, the performance of a grey literature search, and results (see Additional file 2 for a detailed summary table). An appraisal of the methodological quality of the systematic reviews was independently undertaken by two researchers (AG, JL) using A Measurement Tool to Assess Systematic Reviews (AMSTAR) [23]. Discrepancies in quality rating between researchers were reconciled through discussion.

## Results

### Search results

**Fig. 1:**
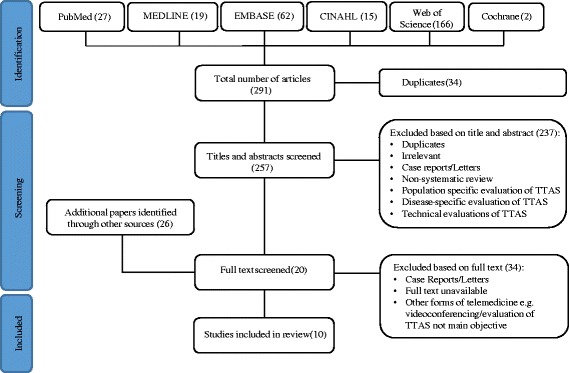
Search flow diagram

A total of 291 papers were identified and subjected to title/abstract review. Of these, 237 were excluded and 20 papers were downloaded and subjected to full text review, along with a further 26 papers identified through hand searching of reference lists. Ultimately, ten systematic reviews were selected for inclusion. Our search flow diagram is depicted in Fig. 1.

### Description of included systematic reviews

The ten selected systematic reviews examined TTAS either alone, or in the context of primary care service models or interventions to improve primary care. The systematic reviews were all published within the period, 2003–2013. Four were published between 2003 and 2006, and six between 2011 and 2013. The reviews came from researchers in the UK, Australia, Canada, the Netherlands and Portugal. Each of these countries had a TTAS service. The UK National Health Service (NHS) established their national TTAS, NHS Direct in 1998, which was replaced with NHS 111 in 2014 [24]. Australia established the healthdirect helpline service in 2006 [25]. Portugal has Health Line 24 [26], and both the Netherlands and Canada have various services. Table 2 details the characteristics and aims of the included systematic reviews.

**Table 2:** Description of reviews

Author and Year	Country	Review Type	Focus of Review	Aim	Included Study Designs	Date Range of Included Studies	Method of Quality Assessment	AMSTAR Quality Rating
Blank et al. [14] 2012	UK	Rapid evidence assessment	TTAS	To synthesise evidence about the appropriateness of and compliance with telephone triage decisions.	52 observational studies 2 RCTs	1992–2010	No documented assessment method	5
Brebner et al. [33] 2006	UK	Systematic review	A & E tele-consultations	To conduct a systematic review of evidence about accident and emergency tele-consultation services.	31 in total; 2 RCTs; mostly demonstration and feasibility studies rather than full-scale trials.	1996–2004	No documented assessment method	4
Bunn et al. [27] 2004	UK	Cochrane Review	TTAS	To assess the effects of telephone consultation on safety, service usage and patient satisfaction, and compare telephone consultation by different healthcare professionals.	5 RCTs 1 CCT 3 Interrupted time series	1979–2002	Modified EPOC checklist (Effective Practice and Organisation of Care Review Group) [29]	8
Carrasqueiro et al. [7] 2011	Portugal	Systematic Review	TTAS	To review TTAS evaluation studies, compile methodologies and metrics used and compare results.	2 qualitative 18 observational 1 decision analysis 31 experimental	1994–2010	Modified economic evaluation checklist	2
Chapman et al. [15] 2004	UK	Systematic review	Recent primary care innovations which included telephone consultations with GPs or nurses	To review the evidence of seven recent innovations (including TTAS-related operations) in service provision to improve access or equity in access to primary care.	4 RCTs 1 Pilot evaluation 1 Lit review 5 observational/descriptive	1993–2003	Hierarchy of evidence assessment, levels 1–3	7
Fry, M. [11] 2011	Australia	Systematic review	After-hours care models	To examine the impact of afterhours primary care models on other healthcare providers, and to examine the effectiveness of after-hours services.	5 RCTs 24 Time series, before and after, or comparative	1985–2009	CASP checklist [31]	4
Huibers et al. [12] 2011	The Netherlands	Systematic review	TTAS	To assess the research evidence on safety of telephone triage in out-of-hours primary care.	33 Total 2 RCTs, 1 case study 30 observational	1989–2011	No documented assessment method	3
Ismail et al. [28] 2013	UK	Systematic review	Primary care interventions (including TTAS)	To review the evidence on primary care service interventions aimed at reducing inappropriate A&E/ ED visits.	6 systematic reviews 13 before and after or interrupted time series 7 cross sectional 6 non-comparative case studies 1 cohort 1 Non-randomised CT	1997–2006	SIGN checklist [32]	5
Leibowitz et al. [30] 2003	Australia	Systematic review	After-hours primary care services including TTAS	A review of the existing evidence about the effects of different models of out-of-hours primary care services on outcome	3 RCTs 2 reviews 1 Evaluation 11 observational/descriptive	1978–2000	Hierarchy of evidence assessment	4
Purc-Stephenson et al. [6] 2012	Canada	Meta-analytic review	TTAS	To investigate the extent to which patients comply with triage advice from tele-nurses and to identify factors potentially affecting compliance.	1 RCT 5 Observational studies 6 Prospective studies 1 Prospective quasi experimental	1997–2010	CASP checklist [31]	6

*TTAS* Telephone Triage and Advice Services, *AMSTAR* A Measurement Tool to Assess Systematic Reviews, *RCT* Randomised Controlled Trial, *CCT* Controlled Clinical Trial, *CT* Controlled Trial, *CASP* Critical Appraisal Skills Programme, *SIGN* Scottish Intercollegiate Guidelines Network, *A&E* Accident and Emergency, *ED* Emergency Department, *EPOC* Effective Practice and Organisation of Care

### Study designs within systematic reviews

Nine of the ten systematic reviews allowed us to identify the studies they included, while one provided an outdated link to their reference list, which proved inaccessible. Our attempts to contact the authors to obtain this information were unsuccessful [7]. From the remaining nine reviews we identified 127 individual papers, 42 (33%) of which were included in more than one review. Thirty-one studies were included in two of the reviews, nine studies featured in three reviews, one study was included in four and another one in five of the reviews.

The studies ranged in date from 1978 to 2011 and originated from the UK (53), the USA (36), Australia (12), Canada (6), The Netherlands and Denmark (both 4), New Zealand and France (both 3), Switzerland (1), Germany (1), Sweden (1), Italy (1), India (1), and Israel (1). There were nine Randomised Controlled Trials (RCTs). The vast majority of studies were observational, including before and after and interrupted time series designs, as well as several surveys. The reviews also included some case studies, evaluations and reports.

### Quality appraisal

To assess the quality of the systematic reviews we: i) conducted a quality appraisal of the systematic reviews against the AMSTAR checklist; ii) evaluated the methodological quality of the studies reported in the review; and iii) considered the comments made in the review regarding the overall quality of the evidence presented.

Out of a maximum score of 11, quality ratings of the systematic reviews against the AMSTAR checklist ranged from 2 to 8. Five reviews achieved a rating greater than or equal to 5 [6, 14, 15, 27, 28]. Three reviews provided no evidence of having assessed the quality of included studies. Of the other seven reviews, a range of methods was used for quality assessment. One used a version of the checklist developed by the Cochrane Effective Practice and Organisation of Care Review Group [29]. Another used a modified checklist for assessing economic evaluations [7]. Two reviews used a hierarchy of evidence method [15, 30] and two reviews used a revised version of the Critical Appraisal Skills Programme [6, 11, 31]. One review used a modified version of the Scottish Intercollegiate Guidelines Network (SIGN) checklist [32]. Eight of the reviews commented on the limitations of the evidence, citing poor study designs, poorly reported studies and low checklist scores. Overall, some of the evidence appears reliable, but much of it is not strong [27].

### Publication bias

Six of the systematic reviews did not include a search of the grey literature [6, 7, 12, 14, 28, 30], while four did [11, 15, 27, 33]. This overview conducted a broad Google search to identify other existing reviews but did not elicit any new results. All searches conducted in this overview were restricted to English language systematic reviews only.

### Quality, safety and governance dimensions

We identified the following nine quality, safety and governance dimensions from the available evidence: access, appropriateness, patient compliance, patient satisfaction, cost, safety, health service utilisation, clinical workload and clinical outcomes. Most of the systematic reviews examined between one to five measures, one review investigated eight, but no review considered all nine. We proceeded to group the review results together based on these indicators and then considered the ways in which they investigated and compared the results.

#### Access

Expanding healthcare access to marginalised communities or populations is an often-cited driver of the introduction or expansion of TTAS services [13]. However, the impact of TTAS on improving access was not apparent from the two systematic reviews that examined this issue. Carrasqueiro et al. considered health care access, but did not state how many studies they found that explored this issue. The authors reported that in some instances healthcare access for some people with severe symptoms was expedited [7], they nevertheless concluded that there was no consistent improvement in access based on TTAS availability. Chapman et al. reported on one study which found that the NHS Direct service was easily accessible, but callers appeared to be those already utilising other healthcare services [15].

#### Appropriateness

In their review, Blank et al. [14] noted the lack of consistency in the definition of “appropriateness” of telephone triage decisions by the studies included in their rapid evidence scan. The five systematic reviews which considered evidence about the appropriateness of TTAS used one of two definitions, examining either the number of calls which could be managed with telephone advice alone, or the appropriateness of the advice given.

Two reviews looked at the percentage of calls able to be handled with telephone advice alone. Both the Cochrane review by Bunn et al. [27] and the review by Fry [11] concluded that approximately 50% of calls handled by doctors or nurses could be handled with telephone advice alone. Bunn et al. also compared the call handling abilities of doctors and nurses and concluded that doctors handled 62% of calls with telephone advice alone, while nurses managed 59% with telephone advice alone [27].

The review by Blank et al. examined studies in which the advice given over the phone was compared with “appropriate” advice – either the “gold standard,” professional advice, subsequent treatment or diagnosis, patient validation or measure of adverse events. This review found a 44–98% rate of accuracy/appropriateness with a median of 75% [14]. The review also compared doctors’ and nurses’ handling of calls, but found that results were not consistent. This review and the review by Fry did however note there may be issues with under-referral and under-estimation of urgency [11, 14].

The review by Leibowitz et al. reported on an RCT which compared the number of deaths in seven days between those whose calls were handled by doctors or nurses. This RCT found no difference between the two groups. This paper also noted that studies using simulated patients reported variability and inadequacies in the advice given over the phone. Alternatively, studies which used real patients reported that the majority received appropriate advice [30].

The review by Carrasqueiro et al. examined studies in which audits of medical records were conducted to determine the adequacy of the advice given over the phone. The authors concluded that they could not demonstrate high rates of advice appropriateness or adequacy, but did not specify exactly how this conclusion was drawn [7]. The impact of other provider characteristics (e.g., age, length of experience) on the appropriateness of the advice given was not discussed by any systematic review.

#### Patient compliance

When advice is offered to patients via a new medium, such as the telephone, it is imperative to assess patients’ willingness to adhere to the advice. Services that provide advice with which patients are unwilling to comply are not likely to prove worthwhile. The three systematic reviews which investigated compliance all compared compliance rates by the advice given, which they grouped into three categories: emergency or urgent care, office/physician care, and home or self-care. Blank et al. reported that the median rate for compliance with advice to seek physician care was 66%, urgent care 75%, and self-care 77% [14].

The Purc-Stephenson and Thrasher review also found that callers were more likely to comply with advice to use emergency services or self-care than office care, but did not provide figures [6]. This review also commented that patient perceptions and expectations, along with the quality of the provider communication influenced caller compliance [6]. The third review by Carrasqueiro et al. [7] failed to provide compliance rates but did report that compliance varied significantly depending on the advice given, and that the variance was due to patient characteristics – original intention, complaint, age, income. This review, as well as the review by Blank et al. [14], also noted that rates of patient compliance were slightly higher when patient self-reporting data were examined, compared with provider data.

Two of the systematic reviews provided overall compliance rates. Blank et al. estimated an overall compliance rate of between 56 and 98%, with a median of 77% [14] while Purc-Stephenson and Thrasher reported an overall compliance rate of 62% [6]. Both of these reviews included some self-reported data, which may be potentially misleading.

#### Patient satisfaction

Five systematic reviews examined patient satisfaction of TTAS with most reporting satisfactory levels. Ismail et al. reported that their analyses found generally positive patient satisfaction rates, although one of the examined studies found low rates [28]. In their Cochrane review, Bunn et al. found patient satisfaction rates to be no different from other forms of care, and sometimes higher [27]. Fry considered patient satisfaction of the (no longer existing) UK NHS Direct service and found several studies reporting good levels of patient satisfaction [11].

Carrasqueiro et al. likewise found that most studies presented positive levels of patient satisfaction, but they, along with Leibowitz et al. observed that satisfaction rates decreased when patients’ initial expectations were not met [7, 30]. Patients were dissatisfied when telephone-based care represented a barrier to traditional forms of care, for example when patients rang physicians requesting a home-visit but were offered a telephone consultation instead [7, 30].

#### Cost

The findings from the four systematic reviews which examined the costs of TTAS, point to the potential for cost-effectiveness, but also suggest a lack of thorough research. Brebner et al. stated simply that all the studies which examined the telephone services found them to be cost-effective and did not elaborate further [33]. Carrasqueiro et al. found no thorough cost analysis studies [7]. Bunn et al. concluded that there was little cost difference between telephone advice and traditional care, but noted a study in which doctors made telephone advice calls to patients instead of granting same day appointments. In this case, the doctors’ phone bills increased 26% [27]. Finally, Ismail et al. concluded that while some cost reductions were possible, analyses suggested that the likelihood of savings across urgent care were low [28].

#### Safety

Two systematic reviews considered the issue of TTAS safety, primarily by investigating the frequency of adverse events, errors and hospitalisation rates. Carrasqueiro et al. reviewed studies which used patient surveys and medical records and concluded that the evidence suggests that TTAS was reasonably safe [7]. Huibers et al. [12] reported on 13 observational studies which showed that on average triage was safe in 97% of patients. However, 10 studies using high-risk simulated patients showed that on average 46% were safe [12].

#### Health service utilisation

Four systematic reviews investigated changes in health service utilisation brought about by the availability of TTAS. Carrasqueiro et al. reported that no clear pattern of change was evident [7]. The review by Ismail et al. reported conflicting results which produced no clear pattern, but acknowledged that reductions in downstream workloads were a possibility [28].

The systematic review by Chapman et al. included one study finding no change in service utilisation, while another study observed a decrease in use of GP services and an increase in out-of-hours consultations and ED attendances [15]. Two reviews reported a reduction in demand for GP consultations, particularly after-hours [11, 27]. One of these found no change in hospital use [27], while the other reported that ED activity could be reduced by up to two thirds [11].

Bunn et al. [27] reported that when telephone consultation was compared with traditional care, two of four studies reported no difference in the number of ED visits, while two studies reported increases in ED visits, one of which was a statistically significant increase, the other was not. Regarding hospital admissions, telephone consultation by doctors did not lead to any change in admission rates, while a phone service run by clinic clerks did lead to a reduction in admissions at 12 months. Bunn et al. also reviewed three studies which compared one type of health care professional with another [27]. Two RCTs compared nurse telephone advice with advice from a doctor in an out-of-hours deputising service [34, 35], and one Controlled Clinical Trial (CCT) compared telephone advice by a health assistant with telephone advice from a nurse or doctor [36]. All three observed a non-statistically significant rise in visits to the ED in the intervention groups. The two RCTs also examined hospital admission rates and found no changes.

#### Clinical workload

Changes in health service utilisation could also produce workload changes for health professionals. GPs may be especially susceptible to such changes. Four reviews considered how TTAS may affect GPs workloads. All four systematic reviews found evidence that telephone advice services had the potential to reduce GPs workloads [11, 15, 28, 30].

#### Clinical outcomes

Two systematic reviews investigated clinical outcomes and clinical effectiveness. The first concluded that ED tele-consultation was clinically effective [33]. Carrasqueiro et al. reviewed studies which utilised patient survey data to consider clinical outcomes. They determined that there were no long term studies which could lead to a conclusion one way or the other [7].

## Discussion

This systematic overview collated the available evidence related to the governance, safety and quality of TTAS across nine key performance indicators – access, appropriateness, compliance, patient satisfaction, cost, safety, health service utilisation, physician workload and clinical outcomes. Some important indicators, such as the *acceptability* of TTAS were not specifically reported in the reviews we examined, but they may be considered in evaluations of performance conducted by the services themselves, or their funding bodies. These evaluations could potentially provide another source of information about these services.

The indicators of *appropriateness* and *patient satisfaction* received the most attention in the reviews. *Appropriateness* was evaluated in different ways ranging from the adequacy of triage advice, to the ability of doctors and nurses to handle enquiries over the telephone [11, 27]. The majority of reviews that investigated *patient satisfaction* with TTAS reported that it was generally comparable to, or occasionally even greater than satisfaction with traditional care [11, 27, 28].

The systematic reviews which examined *safety* compared the frequency of adverse events, errors and hospitalisation rates, and reported that triage was safe in most cases [7, 12]. However, findings from simulated high risk patients showed that on average 50% of patients received advice that was considered unsafe, presenting a higher risk of adverse events [12]. Safety can be very difficult to measure, as those who are already considered unwell or at risk, may be more prone to adverse events, through no fault of the service. Care should be taken about mistakenly using hospitalisation as a measure of safety. If TTAS provides advice that the caller should attend the hospital, this should be seen as advantageous for the patient’s safety, rather than an adverse event related to the use of the TTAS. A clearer definition of adverse events or safety issues related to the service would be of benefit to further research in this area.

The most reliable findings came from studies about *clinician workload* and *patient compliance.* The reviews which evaluated *clinician workload* reported that there was some potential for telephone triage and advice to reduce clinician workload, particularly for GPs. The reviews which investigated *patient compliance* mostly concluded that callers were more likely to comply with advice to seek emergency care or provide self-care, than advice to visit a GP or other health service [6, 7, 14].

The indicators which revealed the most variable and weakest evidence related to the *utilisation* of TTAS, service *access*, and *costs*. Reviews about *access* and the *cost* of TTAS showed no consistent evidence of improvement or gain [7, 15, 27, 28, 33]. Neither of the two reviews which considered *clinical outcomes* provided strong findings.

Taken as a whole, the available evidence does not provide definitive answers to questions about the quality of care provided, access and equity of the service, its costs and outcomes. The available evidence suggests that there are many interactional factors which can impact on measures of performance [6], and also affect the external validity or generalisability of the research findings [37]. For instance, TTAS is one (albeit very important) organisational model for dealing with out-of-hours care. Other models include individual general practices, primary care centres or even GP cooperatives [38]. These models are not necessarily exclusive to one distinct area or location, quite often they co-exist across municipalities, primary health care networks, regions or nations [39]. Decisions about the operation of TTAS are also influenced by a large number of demographic (e.g., rural and remote), cultural (e.g., language), finance (e.g., health care costs) and governance (e.g., policy priorities) factors [39]. Moreover, many of the quality and safety dimensions of TTAS are shaped by factors related to the integration and accessibility of care (e.g., the organisation of primary care) or technical infrastructure (e.g., patient access to information), specific to a community [40]. This suggests that TTAS safety and quality dimensions are intrinsically linked to properties of a larger and broader system [41].

We have in this overview bought together the available evidence and provided a list of measures by which to evaluate TTAS. There is not a simple yes or no answer to questions about the overall effectiveness and benefit of TTAS. Much of the evidence is contingent on the different circumstances and context of different systems. There remain many gaps in the evidence base. This overview provides a framework which can inform future research directions about TTAS and provide guidance relating to their implementation and expansion.

## Conclusion

The diffusion of new technologies (e.g., telehealth care) has provided additional scope to expand and diversify health care services and provide opportunities for greater consumer engagement [42]. In the face of these developments, it is important that researchers continue to seek answers to the many unresolved questions about TTAS [43], albeit with a renewed and greater diversification of qualitative and quantitative research along with strategies that engage with patients as a means of enhancing the patient-centredness of TTAS [44]. Such research endeavours can inform decision making about the governance, quality and safety of TTAS into the future.

## Additional file

Additional file 1: Search strategy. (DOCX 12 kb)
Additional file 2: Results table. (DOCX 16 kb)

## Abbreviations

A&E: Accident and Emergency
CASP: Critical Appraisal Skills Programme
CCT: Controlled Clinical Trial
CT: Controlled Trial
ED: Emergency Department
EPOC: Effective Practice and Organisation of Care
GP: General Practitioner
PICO: Population, Intervention, Comparison, Outcome
RCT: Randomised Controlled Trial
SIGN: Scottish Intercollegiate Guidelines Network
TTAS: Telephone Triage and Advice Services
WebRCT: Web Real Time Communication
