# Supplementary material for: The quality, safety and governance of telephone triage and advice services – an overview of evidence from systematic reviews
Source: BMC Health Serv Res. 2017 Aug 30;17:614. doi: 10.1186/s12913-017-2564-x (PMC5577663; doi:10.1186/s12913-017-2564-x)
Supplement: Additional file 1: — Search strategy. (DOCX 12 kb) [file 12913_2017_2564_MOESM1_ESM.docx]

**SEARCH STRATEGY**

**Search Terms**

| **No.** | **Term** | **Related Terms** |
| --- | --- | --- |
| 1 | **Tele*** | Remote consultation^1^  Tele-consultation  Tele-nursing^1^  Telemedicine^1^  Telephone^1^  Telecommunications^1^  Hotlines^1^  Videoconferencing^1^  Emergency Medical Service Communication Systems^1^  Cell phones^1^ |
| 2 | **Triage** |  |
| 3 | **Advice** | Referral  Consultation |
| 4 | **After hours/ After-hours** | After-hours care^1^ |
| 5 | **Out of hours/ Out-of-hours** | Out-of-hours care  After-hours care^1^ |
| 6 | **Primary Care** | Primary health care^1^  Primary care nursing^1^  Physicians, primary care^1^ |

^1^ Denotes MeSH term

**Search Limiters**

| **Limiters** | |
| --- | --- |
| **Publication Date** | 1990-present |
| **Language** | English |
| **Availability** | Full text available |
| **Article Type** | Systematic Review |
| **Species** | Human |
