# Supplementary material for: The quality, safety and governance of telephone triage and advice services – an overview of evidence from systematic reviews
Source: BMC Health Serv Res. 2017 Aug 30;17:614. doi: 10.1186/s12913-017-2564-x (PMC5577663; doi:10.1186/s12913-017-2564-x)
Supplement: Additional file 2: — Results table. (DOCX 16 kb) [file 12913_2017_2564_MOESM2_ESM.docx]

**RESULTS TABLE**

| **Systematic Review** | **Governance Dimension** | **Measurement** | **Result** | **Grey Literature Search** | **Quality Assessment** |
| --- | --- | --- | --- | --- | --- |
| Blank et al. [14] | Appropriateness, accuracy | Compared telephone advice with ‘appropriate advice’ | 44-98% accuracy/ appropriateness, median 75% | No | No documented evidence of assessment of scientific quality of included studies. |
|  | Patient Compliance | Patient survey;  Billing or provider data | Overall: 56-98%, median 77%  Compliance with all levels of advice: 25-100% |  |  |
| Brebner et al. [27] | Cost-effectiveness | Not specified | Yes, cost effective | Yes | No documented evidence of assessment of scientific quality of included studies. |
|  | Clinical Effectiveness | Not specified | Yes, clinically effective |  |  |
| Bunn et al. [28] | Adequacy | % calls that can be handled with telephone advice alone | Drs and nurses can handle at least 50% of calls with telephone based advice alone; Drs can handle a higher percentage of calls alone than nurses. | Yes | Quality of included studies were assessed using a modified data collection checklist developed by the Cochrane Effective Practice and Organisation of Care Review Group [29]. |
|  | Savings | Not specified | No difference;  Dr’s phone bills increased when Drs called patients who had enquiries |  |  |
|  | Patient satisfaction | Patient surveys | No different to other health services |  |  |
|  | Safety | Compared frequency of adverse events between telephone consultation and traditional care | No significant difference |  |  |
|  | Health Service Utilisation | GP attendance, routine appointments, out-of-hours contacts, ED attendance | Reduction in GP or home visits; no change in hospital use |  |  |
| Carrasqueiro et al.[7] | Access | Patient survey;  operations data | No consistent improvement | No | Critical assessment of studies performed using a modified checklist for assessing economic evaluation. |
|  | Clinical outcomes | Patient survey | No long term studies; some cases resolve, some improve, some require further care |  |  |
|  | Adequacy, accuracy | Audits and medical record review | Unable to demonstrate high levels |  |  |
|  | Patient Compliance | Patient surveys and provider’s databases | Compliance varied significantly depending on the advice given; variance was due to patient characteristics |  |  |
|  | Patient satisfaction | Patient surveys | Generally positive, but dissatisfied if created a barrier to traditional care |  |  |
|  | Safety | Frequency of adverse events | No major safety events observed |  |  |
|  | Changes in health service use | Patient survey, provider data, trend analysis | No clear pattern |  |  |
|  | Cost analyses | Nil | No thorough studies |  |  |
| Chapman et al. [15] | Safety | Compared frequency of adverse events between telephone consultation and traditional care | No significant difference | Yes | Quality of studies assessed by hierarchy of evidence: only levels I-III were included (RCTs, systematic reviews, controlled trials without randomisation, cohort and case studies, studies exhibiting a large difference in outcome over time or space). |
|  | Access | Not specified | NHS Direct is easily accessible |  |  |
|  | Health service utilisation | GP attendance, routine appointments, out-of-hours contacts, ED attendance | Conflicting |  |  |
| Fry [11] | Adequacy | % calls that can be handled with telephone advice alone | Drs and nurses can handle around 50% of calls with telephone based advice alone. | Yes | Included studies were assessed using CASP [30] critical appraisal checklists. |
|  | Patient satisfaction  (with NHS Direct) | Not specified | Good levels of satisfaction |  |  |
|  | H5ealth service util4isation | GP attendance and ED attendance | May reduce ED activity by 2/3;  Reduction in after-hours GP demand |  |  |
| Huibers et al. [12] | Safe6ty | Frequency of errors, mistakes, harm or near harm, unplanned ED attendance or hospital admission and mortality | 10% unsafe with real patients; 50% unsafe with simulated patients | No | No documented evidence of assessment of scientific quality of included studies. |
| Ismail et al. [31] | Patient satisfaction | Patient surveys | Generally positive;  One study reported 55-90% satisfaction; another reported satisfaction comparable with face-to-face care; another reported it was lower than face-to-face care | No | Included studies were classified by design and rated against SIGN [32] checklists. |
|  | Savings | Not specified | Savings possible, but not likely across urgent care |  |  |
|  | Safety | Compared frequency of adverse events between telephone consultation and traditional care | No significant difference |  |  |
|  | Health Service Utilisation and workload | ED attendance rates | Conflicting;  Reduction in downstream workload |  |  |
| Leibowitz et al.[33] | Appropriateness | Not specified | Majority of advice was appropriate, some inadequacies exist | No | Individual studies were assessed by a hierarchy of evidence. |
|  | Patient satisfaction | Patient questionnaires | Low rates, especially when patients expected a home visit |  |  |
| Purc-Stephenson and Thrasher [6] | Compliance with all levels of advice | Patient survey;  Medical records, provider data | Overall compliance: 62% Patients more likely to comply with advice to seek emergency care, or to provide home or self-care, than advice to seek office care.  Compliance with emergency care and home or self-care advice reasonably high. | No | Included studies were individually scored against CASP [30] checklists. |
